# Educational Review: The Impact of Perinatal Oxidative Stress on the Developing Kidney

**DOI:** 10.3389/fped.2022.853722

**Published:** 2022-06-30

**Authors:** Marissa J. DeFreitas, Chryso P. Katsoufis, Merline Benny, Karen Young, Shathiyah Kulandavelu, Hyunyoung Ahn, Anna Sfakianaki, Carolyn L. Abitbol

**Affiliations:** ^1^Division of Pediatric Nephrology, Department of Pediatrics, University of Miami, Miami, FL, United States; ^2^Department of Pediatrics, Batchelor Children’s Research Institute, University of Miami, Miami, FL, United States; ^3^Division of Neonatology, Department of Pediatrics, University of Miami, Miami, FL, United States; ^4^Interdisciplinary Stem Cell Institute, University of Miami, Miami, FL, United States; ^5^Department of Obstetrics, Gynecology and Reproductive Sciences, University of Miami, Miami, FL, United States

**Keywords:** oxidative stress, preterm birth, kidney development, hypoxia, hyperoxia

## Abstract

Oxidative stress occurs when there is an imbalance between reactive oxygen species/reactive nitrogen species and antioxidant systems. The interplay between these complex processes is crucial for normal pregnancy and fetal development; however, when oxidative stress predominates, pregnancy related complications and adverse fetal programming such as preterm birth ensues. Understanding how oxidative stress negatively impacts outcomes for the maternal-fetal dyad has allowed for the exploration of antioxidant therapies to prevent and/or mitigate disease progression. In the developing kidney, the negative impact of oxidative stress has also been noted as it relates to the development of hypertension and kidney injury mostly in animal models. Clinical research addressing the implications of oxidative stress in the developing kidney is less developed than that of the neurodevelopmental and respiratory conditions of preterm infants and other vulnerable neonatal groups. Efforts to study the oxidative stress pathway along the continuum of the perinatal period using a team science approach can help to understand the multi-organ dysfunction that the maternal-fetal dyad sustains and guide the investigation of antioxidant therapies to ameliorate the global toxicity. This educational review will provide a comprehensive and multidisciplinary perspective on the impact of oxidative stress during the perinatal period in the development of maternal and fetal/neonatal complications, and implications on developmental programming of accelerated aging and cardiovascular and renal disease for a lifetime.

## Oxidative Stress and the Perinatal Period

**FIGURE 1:**
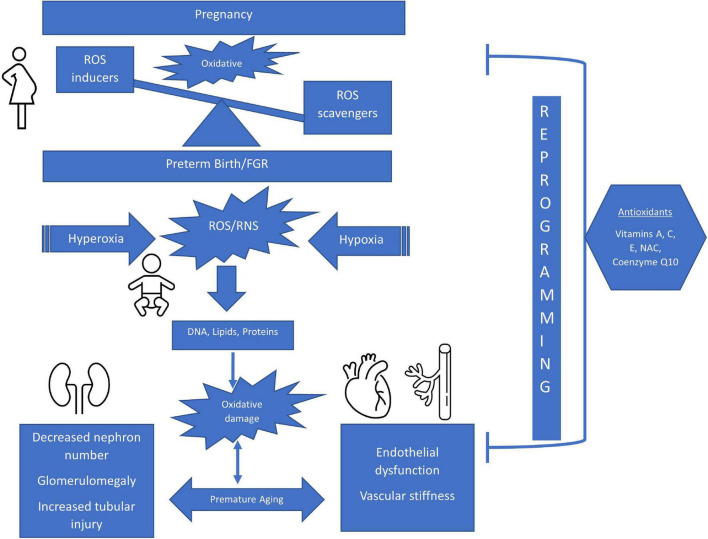
Schematic of The Impact of Perinatal Oxidative Stress on the Developing Kidney and Cardiovascular Systems. ROS, reactive oxygen species; FGR, fetal growth restriction; RNS, reactive nitrogen species; NAC, n-acetylcysteine.

**FIGURE 2:**
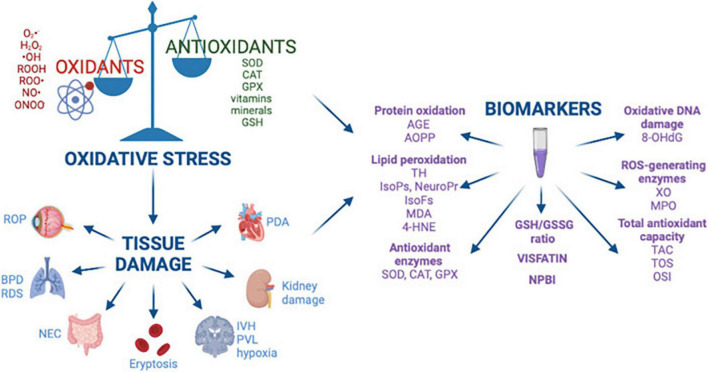
Oxidative stress, free radical mediated diseases of the preterm newborn, and biomarkers. Reprinted from Lembo et al. (3). BPD, bronchopulmonary dysplasia; ROP, retinopathy of prematurity; NEC, necrotizing enterocolitis; IVH, intraventricular hemorrhage; PVL, periventricular leukomalacia; RDS, respiratory distress syndrome; PDA, patent ductus arteriosus; AGE, advanced glycated end product; AOPP, advanced oxidation protein products; TH, total hydroperoxide; IsoPs, isoprostanes; IsoFs, isofurans; NeuroPr, neuroprostanes; MDA, malondialdehyde; 4-HNE, 4-hydroxy-2-nonenal; SOD, superoxide dismutase; CAT, catalase; GPX, glutathione peroxidase; GSH, glutathione; GSSG, glutathione disulfide; NPBI, non-protein-bound iron; 8-OHdG, 8-hydroxy-2′-deoxyguanosine; XO, xanthine oxidase; MPO, myeloperoxidase; TAC, total antioxidant capacity; TOS, total antioxidant status; OSI, oxidative stress index.

Oxidative stress occurs when there is an imbalance between reactive oxygen species (ROS) (1) and reactive nitrogen species (RNS) (2) and the innate antioxidant systems. Under normal circumstances, the perinatal period is propagated by a balanced ROS production for the maternal-fetal dyad. However, in some adverse pregnancy conditions, free radical generation due to the imbalance between oxidants and antioxidants leads to oxidative damage in various organ systems in both the mother and fetus (3). ROS include free radicals, such as superoxide anions which are characterized by highly unstable unpaired electrons and non-radical molecules such as hydrogen peroxide (3). Free radicals are the by-products of metabolic redox reactions in the respiratory chain, microsomal cytochrome P450, and immune response system triggered by several endogenous and exogenous insults such as asphyxia, inflammation, and hyperoxia (4). During pregnancy, a variety of genetic and environmental stimuli can overwhelm the innate antioxidant system allowing the free radicals to react with cellular components and leading to oxidative damage that impacts DNA, protein, lipid and mitochondrial function, ultimately resulting in aberrant fetal programming (5) (Figure 1). The most common ROS include superoxide ion (O_2_⋅-), hydrogen peroxide (H_2_O_2_), hydroxyl radical (⋅OH), hydroperoxide (ROOH), and peroxy radical (ROO⋅) (3). Nitric oxide (NO⋅) is also a key driver of oxidative stress and usually reacts with other ROS to form peroxynitrite (ONOO−), which is part of the RNS. RNS are able to produce similar tissue damage caused by oxidation through the introduction of a nitrogen group into an organic compound (6). Antioxidants, produced endogenously or assumed exogenously, are able to counterbalance free radical production by neutralizing or removing ROS/RNS (6). The most common antioxidants include enzymes superoxide dismutase (SOD) and catalase (CAT), glutathione peroxidase (GPX), vitamins (vitamins C and E), minerals, and small-molecule thiols such as glutathione (GSH) (5). How oxidative stress impacts tissue damage can be evaluated by biomarkers that quantify the levels of oxidation by-products from proteins, lipids, and DNA damage as shown in Figure 2.

## The Intrauterine Environment, Placentation, and Oxidative Stress

The placenta plays an indispensable and multifunctional role as the interface between the two adjoined organisms, the mother and the fetus. It provides an immune interface and serves to transport nutrients and waste products between the mother and the fetus and is a source of peptides and steroid hormones that influence fetal, placental and maternal metabolism and development (7, 8). Human pregnancy is characterized by deep placental invasion, in which extravillous cytotrophoblasts invade into the uterine decidua and the inner third of the myometrium. In normal pregnancy, cytotrophoblasts extend to the spiral arteries in the decidual and myometrial segments and destroy the vascular musculature of the endothelium and the elastic membrane, transforming the arteries into dilated, inelastic vessels without maternal vasomotor control (9, 10). As a result, these vessels are capable of high conductance at low pressure with a low velocity of blood flow entering the placenta. This enables sufficient exchange of nutrients and oxygen through adequate perfusion. It is crucial for placental and fetal development, especially toward the end of pregnancy when fetal demands are highest (11).

In the earliest stage of pregnancy, normal embryogenesis and organogenesis occur in a relatively low-oxygen environment. This physiologic hypoxia in early pregnancy is essential to promote placental angiogenesis, trophoblast cell proliferation and to protect the developing embryo from the teratogenic effects of oxygen free radicals. During this stage, relatively high oxygen tension is toxic to the embryo because of its low antioxidant capacity. As uteroplacental circulation becomes established, the oxygen tension increases in the intervillous space, enhancing the trophoblast invasion for further vascular remodeling (12). There is also an increase in placental mitochondrial mass and mitochondrial electron chain enzyme activity (13, 14) which leads to an increase in ROS production and an increase in oxidative stress. While the placenta provides fetal nutrition and oxygenation, it continuously generates ROS and RNS as the consequences of active oxygen metabolism (14, 15). The increased ROS/RNS is counterbalanced throughout pregnancy by the increased synthesis of antioxidants to maintain homeostasis. To compensate for the increase in oxidative stress, a rise in antioxidant activity (such as glutathione peroxidases and catalases) have been observed as the placenta adapts to the new high oxygen-rich environment (14). Consequently, the embryo becomes more resistant to oxidative stress via improved antioxidant defenses (16, 17). The well-controlled oxidative stress in the placenta plays a role in modulating angiogenesis, immunoregulation, cytotrophoblast invasion, vasoactive function, cellular proliferation, necrosis and apoptosis (18). Disruption of this balance induces inflammatory responses and cellular damage on the developing fetus with teratogenic and long-term consequences, depending on the timing of these events (19–22). Defective deep placentation can lead to uteroplacental insufficiency and chronic placental hypoxia/ischemia, resulting in the adverse outcomes of pregnancy with oxidative stress, especially recurrent pregnancy loss, hypertensive disorders of pregnancy, fetal growth restriction (FGR), gestational diabetes mellitus (GDM) and preterm birth (10, 23).

## Oxidative Stress in Placental Pathologies

When the tight balance between ROS generation and antioxidant generation is interrupted, it can lead to increased generation of chronic oxidative stress leading to inflammatory responses and damage to the cellular system at the DNA and RNA levels (14). This can lead to premature placental aging and pathologies including FGR, preeclampsia (PE), spontaneous pregnancy loss, and GDM (24, 25).

Fetal growth restriction is the failure of the fetus to reach its genetic growth potential (26). It is defined as an estimated fetal weight less than the 10th percentile for gestational age. It is one of the leading causes of fetal, neonatal and perinatal mortality and morbidity. One of the main causes of FGR is believed to be placental insufficiency in early gestation due to abnormal trophoblast invasion in the spiral arteries and the placental bed leading to increased generation of ROS which leads to damage of the placental tissue (24, 27). In FGR placentas, oxidative stress damage is thought to occur predominately in the membrane lipids, proteins and nuclear and mitochondrial DNA. Increased levels of malondialdehyde (MDA) (end products of fatty acid oxidation) and xanthine oxidase (XO) levels have been shown in maternal plasma, umbilical cord plasma and placental tissues of patients with FGR pregnancies suggesting that oxidative stress plays a role in FGR (27). Furthermore, FGR placentas show signs of aging markers, including telomere shortening and absence or reduction in telomerase activity (28, 29). In addition, expression of telomere-induced senescence markers p21 and p16 are increased, while levels of anti-apoptotic proteins Bcl-2 are decreased (28, 30).

Maternal preeclampsia is a life-threatening disorder of pregnancy, characterized by new onset hypertension, proteinuria, abnormal maternal and renal adaptations, poor placenta vascularization and FGR (31). PE affects up to 10% of pregnancies and is one of the leading causes of maternal and fetal/neonatal mortality and morbidity worldwide (31). The etiology of PE remains debatable, but its basic pathology is understood to be vascular endothelial injury mediated by oxidative stress from increased placental ROS and/or decreased antioxidant activity leading to an increase in vascular resistance and a reduction in uteroplacental perfusion (31). In PE, circulating and placental tissue levels of oxidative stress markers such as MDA and 4-hydroxynonenal (HNE) levels have been shown to be elevated and antioxidant (such as catalase and glutathione peroxidase) levels have been shown to be decreased (32–34).

Spontaneous pregnancy loss occurs when the initial onset of the blood flow in the intervillous chamber occurs earlier and is less organized than in normal pregnancies leading to an increase in oxidative stress in the placenta (24).

Gestational Diabetes Mellitus is a serious pregnancy complication, in which women without previously diagnosed diabetes develop chronic hyperglycemia during gestation (35). It is a heterogeneous disorder and involves a combination of factors responsible for decreased insulin sensitivity and inadequate insulin secretion leading to high glucose levels in the maternal blood (35). This hyperglycemic environment is thought to provoke placental oxidative stress and dysfunction, and GDM women have been reported to overproduce free radicals and have impaired free-radical scavenging mechanisms (35).

As described above, uteroplacental insufficiency from defective placentation can provoke chronic hypoxia, leading to PE, FGR, neurodevelopmental delay and intrauterine fetal death. Hypoxia can cause tissue damage via severe oxidative stress. Recently, several experimental studies demonstrated the beneficial effect of antioxidant administration via NO-dependent mechanisms. In pregnant sheep, Melatonin or vitamin C increased fetal umbilical blood flow through NO synthase-dependent vasodilation (36). In pregnant mice, N-acetylcysteine (NAC) decreased the cadmium (Cd)-induced placental insufficiency and FGR (37). In addition, in pregnant mice lacking a denitrosylase, S-nitrosoglutathione reductase, vitamin C treatment rescued the maternal PE-like phenotype, including hypertension, proteinuria, renal pathology, cardiac concentric hypertrophy and decreased placental vascularization (38). In humans, several studies have demonstrated that NO donor improved maternal and fetal hemodynamics in PE and FGR (39–42). However, the current studies are limited due to the small sample size and the observational nature, although they demonstrate the potential effectiveness of antioxidant therapy.

## Oxidative Stress and Programming of Nephron Number and Kidney Injury

Nephrogenesis in humans is intended to be complete by 36 weeks’ gestation *in utero* (43, 44). Preterm infants are born during active nephrogenesis, making them particularly vulnerable to alterations imposed by the extra-uterine environment (45, 46). The preterm-born individual’s kidney function will depend on the effective nephron mass, which is proportional to the number of perfused and fully formed glomeruli (47). The kidneys form in parallel with other organ systems by a process of branching morphogenesis (48–50). Among the organ systems that develop by branching morphogenesis are the lungs, pancreas, vascular tree and kidneys, which share genetic and physiologic functional determinants within the fetal origins of adult disease paradigm (51–53).

In several animal models of renal programming, maternal malnutrition/diet, diabetes and steroid exposure have been linked mainly to the development of hypertension in the offspring, and have been associated with an increase in oxidative stress through various mechanisms (5). These studies have been enumerated in Table form by Hsu et al. (5). In addition, some animal studies have reported specifically on a reduced nephron number associated with increased oxidative stress in offspring after a maternal insult (5). In particular, this has been reported in a caloric restriction model (54), streptozotocin-induced diabetes (55), and maternal smoking (56). Tain et al. showed that ureteric bud branching morphogenesis was inhibited by asymmetric dimethylarginine (ADMA), a ROS inducer and endogenous NOS inhibitor, leading to decreased nephron number (55). However, some studies have not shown a decreased nephron number but rather glomerular hypertrophy (54, 57–60) and tubulointerstitial injury (54, 55, 57, 59–61) as the renal phenotype associated with oxidative stress and perinatal programming. The main mechanisms of oxidative stress shown in these studies to be associated with renal injury include increased levels of asymmetric dimethylarginine (ADMA), F2 isoprostane, renal 8-hydroxydeoxyguanosine (8-OHdG) and MDA and decreased levels of NO and superoxide dismutase (62).

If birth occurs prematurely during active nephrogenesis, there may be potential for ongoing or even accelerated nephrogenesis; however, the often hostile extrauterine environment with various oxidative stressors likely limits this window of opportunity. However, our knowledge of ongoing nephrogenesis in preterm humans is limited to a few postmortem studies, showing abnormalities in postnatal nephrogenesis in preterm born individuals (45, 46, 63). Rodriguez et al. showed that a full complement of nephrons was never achieved in preterm as compared to term newborns and that acute kidney injury (AKI) led to further restriction of nephron endowment (45). In addition, others have noted a high percentage of morphologically abnormal glomeruli, including atubular and cystic glomeruli, which would not be able to function as well as high interindividual variability in radial glomerular counts and glomerular morphology in postmortem studies of preterm infants (46, 63). Importantly, a low nephron endowment has been linked to the development of hypertension and cardiorenal disease in adult life (64).

## Accelerated Aging and Oxidative Stress in Preterm Infants

Preterm infants are known to have a shortened lifespan and acceleration of aging in large part due to cardiovascular disease, diabetes mellitus and chronic kidney disease (CKD) (65). Evidence of accelerated aging has been noted in preterm infants both early and in later life (66–68). Oxidative stress is a driver of the natural aging process (69) and, as such, functions as a therapeutic target. Leukocyte telomere length is a biomarker of aging-related health risks (70, 71). Hospitalized preterm infants frequently experience elevated oxidative stress and inflammation, both of which contribute to telomere shortening. Belfort et al. studied changes in telomere length during neonatal intensive care unit (NICU) hospitalization in a cohort of preterm infants (71). They noted that from birth to discharge, preterm infants (mean gestational age 27 weeks, range 23.5–29 weeks) experienced a significant weekly decline in relative telomere length (71). Luyckx et al. showed in a rodent model that low-birth-weight rats exhibited accelerated senescence in kidneys and hearts after rapid catch-up growth (72).

## Postnatal Oxidative Stress Induced Kidney Injury and Emerging Biomarkers

In the developing kidney, the negative impact of oxidative stress related to fluctuating oxygen exposure from hyperoxia to intermittent hypoxia, has also been implicated in the development of kidney injury in postnatal animal models (60, 73–78) (Table 1). Nephrogenesis is complete by the 36th week of gestation in humans, but it continues until approximately postnatal day 10–14 in rats (73). Hence, the various exposures to oxygen in neonatal rats could have comparable consequences to those seen in extremely premature infants during the critical period of postnatal nephrogenesis, which is limited to 40 days and occurs aberrantly (45, 63, 79). Experimental and early clinical studies are now emerging that oxygen exposure in these preterm babies can lead to vascular and renal axis alterations, leading to hypertension, stroke and renal failure into adulthood (62, 80–82). The discovery of biomarkers to better understand the role of oxidative stress in early postnatal kidney injury is critical to allowing for the development of translational studies aimed at developing targeted therapies.

**TABLE 1:** Rodent studies of hyperoxia and/or hypoxia related renal injury during postnatal nephrogenesis.

Study reference	Year of publication	Animal	Oxygen exposure	Sacrifice	Glomerular size	Tubular injury	Fibrosis	Nephron number
(77)	2008	Rat	80% P3-P10	25–35 weeks	NA	NA	NA	Decreased by 25%
(76)	2013	Mouse	80% P3-P10	P5 and P10	P5 decreased P10 no change	NA	NA	Not affected
(74)	2013	Rat	65% × 1 week	P7 and P56	P7 no change P56 increased 10 months no change	NA	NA	Not affected
(78)	2015	Rat	95% × 1 week 85% × 2 weeks	P7 and P21	P7 and P21 increased	P7 and P21 increased	Increased	NA
(73)	2016	Rat	80% P3-P10	1 month, 5 months 11 months	No change at any time point	NA	No change	Not affected
(75)	2020	Rat	85% P1-P21→ 21% × 3 weeks	P42	Increased	Increased	NA	NA
(79)	2021	Rat	50% with IH 12% w/varying exposure durations	P7, P14, P21	Increased	Increased	NA	Decreased

*P, postnatal day; IH, intermittent hypoxia; NA, not applicable.*

Advanced oxidation protein products (AOPPs) are an established universal biomarker of oxidative injury. AOPPs are crosslinked protein products formed during oxidative stress by the reaction of plasma protein with chlorinated oxidants. Accumulation of plasma and renal AOPPs is a common pathologic finding in chronic kidney disease (CKD) patients and is an independent risk factor for cardiovascular events in CKD (83). AOPP have been studied in neonatal oxidative injury and reference values established in a healthy population of term newborns to allow for comparison in preterm groups (84). Few studies have looked at the role of AOPPs in neonatal oxidative stress related kidney injury *in vivo*. Perrone et al. investigated 55 preterm infants exposed to perinatal hypoxia and found increased AOPP in the first 14 days of life as expression of oxidative stress-induced cellular damage (85).

Alpha-Klotho is a transmembrane protein highly expressed in the kidney, and its cleaved product in the circulation (soluble Klotho) functions as an endocrine hormone with potent antioxidant and antifibrotic properties (86). CKD is a state of Klotho deficiency that exerts multiple systemic adverse effects, including the vascular system (86–89). Our group demonstrated in a rodent model of hyperoxia-induced kidney injury that hyperoxia exposure during early postnatal nephrogenesis was accompanied by a marked reduction of renal Klotho expression, restricted renal perfusion, and glomerulomegaly and tubular injury (60). Administration of exogenous Klotho improved renal vascular perfusion, abrogated glomerulomegaly and tubular injury, and restored kidney antioxidant capacity [manganese superoxide dismutase (MnSOD) and catalase mRNA expression] (60).

An experimental model of renal insufficiency in adult rats showed that early administration of α-Klotho prevented the progression of AKI to CKD and protected the heart from cardiac remodeling (90). The supplementation of exogenous Klotho and/or upregulation of endogenous renal Klotho production may confer dual renal and lung protection, which is proposed to be associated with, but not restricted to, its antioxidant properties (91, 92). Our group also showed that umbilical cord Klotho levels were depressed in infants with bronchopulmonary dysplasia (BPD) and pulmonary hypertension and that Klotho administration improved pulmonary hypertension, left ventricular hypertrophy and cardiac dysfunction in the same hyperoxia rat model (93). Future studies should be directed toward understanding the molecular pathways driving oxidative stress and injury, as well as looking at the comparison between early and late outcomes of these on the developing kidney. Therapeutic strategies to prevent neonatal AKI and progression to CKD are lacking. Our findings call for future translational studies to explore the clinical applications of exogenous Klotho administration and/or therapies that promote endogenous Klotho production in premature infants with oxidative stress-related renal injury.

Experimental and clinical data suggests that increased Plasminogen Activator Inhibitor-1 (PAI-1), a marker of oxidative stress and aging, is associated with cardio-renal dysfunction. It is now well-recognized that natural aging is associated with a decline in GFR related to a loss in nephron mass over time due in part to progressive renal scarring (94). PAI-1 has been implicated in the mechanism of renal scarring seen during aging (94). PAI-1 is a member of the superfamily of serine protease inhibitors and plays a major physiologic role in inhibiting tissue type plasminogen activator (t-PA) and urokinase type plasminogen activator (u-PA), which both activate plasminogen to plasmin thus promoting fibrinolysis. PAI-1 also assists with matrix degradation by regulating t-PA and u-PA, which are both fibrinolytic. Hence, upregulation of PAI-1 leads to accumulation of extra cellular matrix because t-PA and u-PA are not allowed to proceed with normal fibrinolysis. Angiotensin II is a promoter of PAI-1 and recently it was shown in rats that use of angiotensin receptor blockers slowed the progression and even led to regression of glomerular and vascular sclerosis in aging through inhibition of PAI-1 expression (95). In addition, investigators were able to show that transferring bone marrow derived cells from young to old mice resulted in alleviation of renal aging by decreasing cellular senescence while reducing PAI-1 activity (96). How PAI-1 activity is altered by preterm birth and postnatal oxygen exposure or whether it can serve as a biomarker of early cardio-renal disease in this population in unknown.

## Organ Cross Talk and Prematurity Related Oxidative Injury

### Lung-Kidney

Bronchopulmonary dysplasia (BPD) is a leading cause of morbidity and mortality in preterm infants. Evidence from preclinical studies demonstrate that preterm animals have blunted antioxidant response to hyperoxia and exposure to supraphysiologic postnatal oxygen levels was originally thought to be a leading cause of BPD (97). More recent data suggest that BPD is a multifactorial disease originating prior to delivery. Suboptimal intrauterine conditions along with adverse neonatal exposures during a pivotal period of organogenesis increase oxidative stress and inflammation, with damage to proteins, carbohydrates, DNA and lipids. Activated inflammatory cells release free radicals, creating a vicious cycle that disrupts intercellular communication, induces cellular apoptosis and promotes organ injury (98). Interestingly, preterm infants with AKI have a higher risk of BPD (99–101). Most preterm infants who develop BPD are in the cannalicular to saccular stage of lung development. Like the kidney, the lungs develop by a process of “branching morphogenesis” (44, 51, 52) and share genetic and physiologic functional determinants, suggesting potential dysregulation of overlapping signaling pathways by prematurity and its associated insults.

High levels of oxidative stress markers, such as lipid or protein oxidation products in the cord blood, plasma and bronchoalveolar lavage fluid of preterm infants who develop BPD suggests that oxidative stress plays a significant role in the pathogenesis of BPD. Elevated lipid peroxidation products, advanced oxidation protein products and non-protein bound iron in cord blood is associated with increased risk of the preterm free radical-related diseases, including BPD (102).

### Cardiovascular-Kidney

Preterm birth occurs during a key period of cardiovascular development, where the premature heart is exposed to the rapid transition from a low resistance placental circulation to a high resistance, high flow systemic circulation. The heart grows *in utero* by increasing the number of cardiomyocytes until term. Preterm birth leads to an abrupt reduction in cardiomyocyte proliferation (103). There is also an increase in the left ventricular mass by the first month of life suggesting marked myocardial adaptation to the extra-uterine environment (104). Evidence from animal models and clinical studies of premature infants show that preterm birth interferes with normal cardiac development with consequences beyond childhood and into adulthood. A recent meta-analysis shows that preterm-born individuals have morphological and functional cardiac impairments across developmental stages from birth to adulthood (105). Cardiovascular magnetic resonance of preterm-born young adults showed that these individuals had a greater left ventricular (LV) wall thickness and mass, and smaller LV end diastolic volumes, LV cavity dimensions and length (106).

In animal models, postnatal hyperoxia induces cardiomyocyte cell-cycle arrest through activation of the DNA damage response, while scavenging ROS or inhibiting DNA damage delays cell cycle arrest (107). Moreover, on long-term follow up, transient oxygen exposure enhances fibrosis, increases oxidative stress, increases expression of senescence-associated proteins and augments susceptibility to heart failure under pressure overload (93, 108). Preterm birth may disrupt or even prematurely arrest the development of the vasculature, impacting the vessel structure (109). Elastin, the scleroprotein that imparts distensibility to the large vessels is accrued during the last trimester of rapid vasculogenesis. Collagen, the structural protein that imparts rigidity to the vessel, accrues at a slower rate *in utero*. At term, the vascular wall contains at least 60–80% elastin and less collagen. The turnover of elastin is extremely slow, with a half-life of 40 years and almost no appreciable turnover of elastin in the adult aorta (110). Thus, a disruption of elastin synthesis due to preterm birth is poised to have long term consequences (111).

Oxidative stress and high oxygen exposure after birth may lead to the increase of ROS and cause endothelial dysfunction, a well-recognized marker of cardiovascular disease (112, 113). There is evidence that neonatal hyperoxia exposure increases ROS production and alters elastin and collagen distribution within the systemic vasculature (112–114). Rat pups exposed to neonatal hyperoxia demonstrated increased collagen to elastin ratio at 4 weeks (114). This was accompanied by increased aortic pulse wave velocity (PWV), a marker of vascular stiffness when the rats were evaluated at 6 and 9 months, suggesting that neonatal hyperoxia plays a crucial role in the pathogenesis of systemic vascular dysfunction in adults born preterm (62, 80, 82, 115, 116). Evidence from epidemiological studies shows a significant inverse correlation between systemic blood pressure and gestational age at birth, which is consistently observed from childhood to adulthood in preterm born adults (117–119). In addition, young adults born preterm have reduced size of the ascending aorta and increased stiffness of the brachial and carotid arteries (120). In the HAPI study (Health of Adults born Preterm Investigation), young adults born preterm had smaller kidneys, higher urine albumin/creatinine ratios, higher angiotensin I levels, and higher blood pressure compared to those born full-term (121).

## Antioxidant Therapies Targeting Minimization of Neonatal Kidney Injury

As delineated above, perinatal oxidative stress is generated in both environments of hypoxic ischemia as well as hyperoxia, with excessive ROS associated with the downstream impact on reduced nephron number and renal fibrosis as well as parallel injury in the lungs, heart and vascular systems (122). As such, the imbalanced increase in ROS may be amenable to antioxidant therapy which, if administered in the perinatal period, may impact the development of adult chronic diseases. Antioxidants may be categorized as enzymatic or non-enzymatic, natural or synthetic. By mechanism of action, they are classified as: suppressants of radical formation, radical scavengers, or repair agents of molecular damage (123). While no agent is currently recommended for routine administration by clinical practice guidelines, we recognize the potential benefit of antioxidant therapy through the current evidence in pre-clinical animal models, limited clinical trials, and the study of patients with advanced CKD.

### Vitamins

Vitamins C (ascorbic acid) and E (α-tocopherol) are scavengers of free radicals. Vitamin E specifically interferes in lipid peroxidation, while Vitamin C facilitates the recycling of Vitamin E (124). When administered perinatally, they have been shown to prevent the development of hypertension and kidney injury in the adult offspring of the spontaneously hypertensive and Fawn hooded hypertensive rat models, respectively (5). Furthermore, clinical trial in a dialysis-dependent population revealed less cardiovascular disease associated with decreased biomarkers of oxidative stress (125). However, the same was associated with an increase in all-cause mortality in adults with chronic diseases, and with prostate cancer in otherwise healthy men (5).

### Amino Acids

Renoprotection has been shown with the exogenous administration of antioxidant amino acids. L-taurine in combination with other antioxidants can circumvent the development of hypertension, proteinuria, and glomerulosclerosis in genetic hypertensive rat models (126). In addressing NO deficiency, L-arginine (the substrate for NO) and its precursor L-citrulline have shown beneficial effects in animal models of renal programming but remain inconclusive in clinical trials in adults (5).

### N-Acetylcysteine

N-acetylcysteine (NAC) is a well-known scavenger of free radicals that also increases antioxidant capacity as a precursor to glutathione (127). While evidence in the clinical studies of adults has been inconclusive, its potential role in neonatal kidney disease is strengthened by multiple experimental models. Beneficial effects against postnatal oxidative stress have been seen in a rat model of sepsis (128), in a porcine model of neonatal asphyxia (129), and in a clinical trial in neonates undergoing cardiac surgery for congenital heart disease (130). Of specific interest for practical application, it is noteworthy that therapeutic administration was post-injury in the above-mentioned rat and porcine studies. This is compared to the pre/peri-injury administration in many other experimental models. Furthermore, the addition of Vitamin D to NAC is proposed to have a synergistic increase in glutathione concentrations and is currently under study (NCT04643801) (131).

### Coenzyme Q10

Given its high reliance on aerobic metabolism and thereby on mitochondrial function, the kidney is dependent on Coenzyme Q10 (CoQ10) in its electron-shuttling action (132). CoQ10 can further reverse mitochondrial dysfunction by preventing phospholipid peroxidation and free radical oxidation, as it has been shown to do in an intermittent hypoxia rat model designed to replicate the variable oxygen tension exposure of preterm infants (132). In a maternal smoking mouse model, it protected adult offspring from hypertension, kidney injury, and oligonephronia (56).

### Melatonin

Melatonin is an endogenous indolamine neurotransmitter that has multiple neurohormonal functions including serving as a scavenger of ROS and an upregulator of antioxidant enzymes (133). As such it has been given exogenously as an antioxidant therapy in a variety of conditions including during the perinatal period for both mother and infant (133, 134). Reduced levels of melatonin are found in women with preeclampsia (135). Melatonin has been shown to regulate blood pressure and mitigate the development of hypertension in numerous animal models of renal programming (3). Recently, an increased urinary angiotensinogen/melatonin ratio was suggested to be an early biomarker for identification of gestational diabetes or pregnancy induced hypertension (136). In neonates, there is placental transfer of melatonin as endogenous melatonin production does not occur until 2–4 months of age and is known to be further delayed in preterm and FGR infants (134). Studies in animals have demonstrated the usefulness of melatonin in preventing and reducing cerebral inflammation in cases of perinatal hypoxic damage and during fetal ischemia and reperfusion (135). Several clinical studies using melatonin showed that it ameliorates oxidative stress in newborns with sepsis, asphyxia, or other conditions where there is excessive ROS production (133). Additionally, the urinary excretion of a melatonin metabolite has been reported to be impaired in adults who were growth restricted prenatally or were born after 40 weeks of gestation, suggesting potential fetal programming of melatonin production that may impair melatonin related pathways in adulthood such as antioxidant defenses (135). Finally, direct nephrotoxicity from compounds such as cisplatin have been shown to be mitigated by melatonin given its antioxidant properties (137). The role of melatonin in neonatal oxidative stress related kidney injury has not been fully explored.

## Other Oxidative Stress Lowering Therapies to Avert Chronic Kidney Disease Progression

The kidney is among the fastest aging organs and expression of the senescence marker p16 has been shown to correlate best with renal aging, though it has not been studied in association with birth weight (138). Several studies have been conducted to understand the mechanisms behind oxidative stress, renal aging and the progression of CKD (139). Investigations into therapeutic discoveries that can slow the progression of CKD in adult and animal studies can potentially play a role in prolonging kidney function in prematurity-related kidney disease as well.

Blockade of the renin-angiotensin system (RAS) is now standard of care to slow the progression of proteinuric CKD (140). Angiotensin receptor blockers (ARB) have been shown to have anti-inflammatory and anti-oxidative properties in both cardiac hypertrophy and various kidney injury models including obesity and diabetes (141). For example, in obese rats, ARB treatment showed anti-inflammatory and anti-oxidative properties with reduction in heme oxygenase-1 (141, 142). Another experimental model of diabetic kidney injury, showed that ARB reduced albuminuria and was associated with increased NO production (143). However, during fetal and early renal development, the RAS system is instrumental in driving normal kidney development (44). In fact, exposure *in utero* to RAS inhibitors such as angiotensin receptor blockers or angiotensin converting enzyme inhibitors from mothers who are hypertensive or have CKD, leads to RAS fetopathy heralded by renal dysplasia and severe renal insufficiency, along with extra-renal abnormalities (144). As such, use of RAS inhibition is generally avoided during the first 2 years of life, until the kidney is fully matured (145). Hence, utilizing RAS inhibition in prematurity-related kidney disease before 2 years of life is limited. Nonetheless, RAS activation is known to drive oxidative stress and fibrosis in kidney disease (146).

Experimental studies have shown that sodium-glucose cotransporter 2 (SGLT2) inhibitors have diverse effects including modulation of the RAS as well as attenuating systemic inflammation and oxidative stress, which has been previously associated with cardiorenal protection in patients with type 2 diabetes mellitus (147). Given the significant improvement seen in CKD progression, SGLT2 inhibitors are now recommended for CKD with albuminuria regardless of the presence of diabetes, as it has been shown to slow estimated glomerular filtration rate (eGFR) decline, delay the onset of end stage kidney disease (ESKD), and decrease all-cause mortality (148). SGLT2 inhibitors are thought to reduce oxidative stress in the kidney by decreasing intracellular glucose in proximal tubular cells (147). How SGLT2 inhibitors improve renal outcomes outside of diabetes is incompletely understood. In addition, lipid dysmetabolism is implicated in oxidative kidney damage in diabetic and non-diabetic forms of CKD. Currently, the literature suggests that both quality and quantity of lipids contribute to increased ROS production, oxidative stress, inflammation, cell death (149). Unraveling the complex nature of lipid dysmetabolism and which lipid species are most important in progression of kidney disease will help with targeted drug therapy development. Translation of findings in drivers of CKD progression in animal and adult models to experimental models and clinical studies of prematurity-related kidney disease will help fast track therapeutic discovery.

## Supporting Infant Growth and Nephron Health in Early Childhood, Minimizing Accrual of Oxidative Insults

The perinatal programming of kidney health is largely determined by genetic and maternal risk factors with induction of oxidative stress through pathogenic pathways that lead to reduced nephron endowment associated with FGR, PE, maternal nutritional deficiencies and preterm birth (150–152). After birth, there is a critical window in which the nutritional and postnatal environmental exposures can influence nephrogenesis. Growth trajectories and neonatal nutrition are known to contribute to the developmental programming of nephron endowment, insulin resistance and cardiovascular and renal disease in later life (153–155).

Accelerated postnatal growth in preterm infants, whether small or appropriate for gestational age, contributes to the precocious development of the metabolic syndrome with insulin resistance in early childhood, which is further confounded by excessive adiposity (155, 156). This, in turn, contributes to the early progression of CKD, especially in those born preterm with low nephron endowment (157–159).

The early neonatal nutritional prescription is of paramount influence on kidney development and will be a major determinant of cardiovascular and renal health for the entire life course. The macronutrient and micronutrient (vitamin and mineral supplements) distributions require close review and planning (155, 157). The adverse long-term effects of perinatal exposure to excessive carbohydrate energy, low protein diet, high fat diet, high fructose diet, high and low salt diets, as well as micronutrient deficiencies have been documented in human and laboratory studies (155, 157). These effects are established during “developmental time slots” when the system is primed for staged development during gestation and the perinatal period. As such, specific alterations in the nutritional environment may program for nephron endowment, blood pressure dysregulation, endothelial and mitochondrial dysfunction. Much of these programmed effects are mediated by oxidative stress and metabolic pathways which are perpetuated throughout the lifetime of the individual. The classic example is that of the epidemiologic observations of individuals exposed during gestation to maternal protein-calorie malnutrition during the Dutch (1944/1945) and Chinese (1959–1961) famines resulting in adult hypertension, proteinuria and metabolic syndrome/obesity (160, 161). Isolated protein malnutrition in animal and human models is well-recognized to result in smaller kidneys and lower nephron numbers (162–164). Subsequent accelerated growth, in response to hypercaloric intake, results in obesity, proteinuria, hypertension and accelerated senescence and progression of CKD (162–164). Carbohydrates are the major macronutrients and provide the bulk of the calories at 45–50% in most diets including infant formulas and parenteral nutrition. The other 2 macronutrients are protein at 10–20% and fat at 20–30%. The issue is complex, but the basic tenet is to examine the distribution of each of the macronutrients followed by the best fit of formula for the infant’s phenotype.

Micronutrients include vitamins and minerals that are essential to growth and health maintenance. In a well-balanced diet, supplementation is rarely required. However, in the setting of preterm birth, FGR, PE and other disorders of oxidative stress, modulation, restriction and/or supplementation may be beneficial. Excessive salt intake in mothers and infants may result in elevated blood pressures and dysregulation of the RAS system. Low levels of the antioxidant vitamins including vitamins A, C, E, and folate have been associated with low nephron endowment, hypertension and decreased longevity (157, 163).

The follow-up of infants with predisposition to CKD including those with extreme preterm birth, neonatal AKI, FGR, gestational hypertensive exposure, twin gestation and congenital anomalies of the kidney and urinary tract (CAKUT) should include monitoring of the diet, avoidance of excess salt, hypercaloric formulas and overweight/obesity. Adequate micronutrient supplementations are indicated, in addition to supplemental antioxidants, in conditions of suspected mitochondrial dysfunction and periods of oxidative stress.

## The Importance of Team Science and Translational Study Design in the Developmental Origins of Cardio-Renal Disease Framework

The concept of team science has evolved over the last two decades. Team science is a collaborative and cross-disciplinary approach to scientific inquiry that draws researchers, who otherwise would work independently or as co-investigators on small scale projects, into collaborative centers and groups (165). The developmental programming of health and disease model lends itself to formulating collaborative team research since many of the overriding insults, such as oxidative stress, have global implications for the maternal-infant dyad. As such, translational research projects could be more effective in targeting mechanisms and drug discovery for this vulnerable population. This model has been embraced for example in Portugal based off the European Respiratory Society Research Seminar “Early origins of lung disease: toward an interdisciplinary approach” where they describe that future research into early origins of lung disease should be centered around four major focus areas: (1) policy and education, (2) clinical assessment, (3) basic and translational research, and (4) infrastructure and tools. The authors acknowledge that interdisciplinary funding opportunities are scarce but an important opportunity to move the field forward dependent on the development of biospecimen repositories (166). The International Society for Developmental Origins of Health and Disease Society (DOHaD) is an organization that fosters multidisciplinary exchange of ideas centered around this developmental model while promoting team science.

## Summation

In conclusion, oxidative stress plays a role in the physiologic transition from intrauterine to extrauterine life and can contribute to significant pathology in the maternal-infant dyad when it goes unbalanced. The downstream inflammation and cellular damage that results from oxidative stress has implications on the developing kidneys, lungs, and cardiovascular systems. Use of antioxidant therapies to mitigate impaired nephrogenesis as well as development of other key organ systems have shown promising results largely in animal models and future studies call for the translation of this work into clinical investigations. The use of a team science approach and building of biorepositories for study of the maternal-infant dyad can help to organize the discovery and antioxidant therapies with potential global benefit for mother and baby.

The implication of ROS in multiple neonatal morbidities, referred to as “oxygen radical disease of neonatology” (167), has more recently extended to include the kidney, both postnatal and developmental origins of adult CKD. The transition to the extrauterine environment is one marked by oxidative aggression, particularly for the preterm infant with impaired antioxidant capacity (168). Because oxidative stress is implicated in the pathophysiology across multiple organ systems, it is of vital importance to build upon the current evidence to develop clinical practice guidelines for antioxidant therapy in this vulnerable and expanding population.

## Author Contributions

MD, CK, MB, KY, SK, HA, AS, and CA contributed to the manuscript design, production, and editing and approval process. All authors contributed to the article and approved the submitted version.

## Conflict of Interest

The authors declare that the research was conducted in the absence of any commercial or financial relationships that could be construed as a potential conflict of interest.

## Publisher’s Note

All claims expressed in this article are solely those of the authors and do not necessarily represent those of their affiliated organizations, or those of the publisher, the editors and the reviewers. Any product that may be evaluated in this article, or claim that may be made by its manufacturer, is not guaranteed or endorsed by the publisher.
